# Wogonin Attenuates Ovalbumin Antigen-Induced Neutrophilic Airway Inflammation by Inhibiting Th17 Differentiation

**DOI:** 10.1155/2014/571508

**Published:** 2014-05-28

**Authors:** Rie Takagi, Masaaki Kawano, Kazuyuki Nakagome, Kumiko Hashimoto, Takehiro Higashi, Katsuya Ohbuchi, Atsushi Kaneko, Sho Matsushita

**Affiliations:** ^1^Department of Allergy and Immunology, Faculty of Medicine, Saitama Medical University, Saitama 350-0495, Japan; ^2^Department of Respiratory Medicine, Saitama Medical University, Saitama 350-0495, Japan; ^3^Allergy Center, Saitama Medical University, Saitama 350-0495, Japan; ^4^Tsumura Research Laboratories, Tsumura & Co., Ibaraki 300-1192, Japan

## Abstract

Allergic airway inflammation is generally considered to be a Th2-type immune response. Recent studies, however, have demonstrated that Th17-type immune responses also play important roles in this process, particularly in the pathogenesis of neutrophilic airway inflammation, a hallmark of severe asthma. We scrutinized several Kampo extracts that reportedly exhibit anti-inflammatory activity by using *in vitro* differentiation system of human and mouse naïve T cells. We found that hange-shashin-to (HST) and oren-gedoku-to (OGT) possess inhibitory activity for Th17 responses *in vitro*. Indeed, wogonin and berberine, major components common to HST and OGT, exhibit Th17-inhibitory activities in both murine and human systems *in vitro*. We therefore evaluated whether wogonin suppresses OVA-induced neutrophilic airway inflammation in OVA TCR-transgenic DO11.10 mice. Consequently, oral administration of wogonin significantly improved OVA-induced neutrophilic airway inflammation. Wogonin suppressed the differentiation of naïve T cells to Th17 cells, while showing no effects on activated Th17 cells.

## 1. Introduction

Different classes of specific immune responses are driven by the biased development of effector CD4^+^ T-cell subsets, that is, T helper 1 (Th1), Th2, and Th17 cells, that activate different components of cellular and humoral immunity. Th-cell differentiation is critical for achieving proper immune responses, and imbalances in either the function or activity of these cell types are responsible for many immune diseases, including autoimmunity, cancer, and allergies [1–3]. T-cell receptor stimulation and costimulation allow naïve T cells to develop into protective effector cells, normally accompanied by the high-level expression of selective sets of cytokines. The balance between these cytokines and the resulting class of immune responses strongly depends on the conditions under which the DCs are primed for the expression of T-cell-polarizing molecules [4].

We recently found that such activities can be scrutinized by assessing mixed lymphocyte reactions (MLR) [5], cAMP [6], and the differential expressions of Notch ligand isoforms [7]. Of note, MLR meets the needs for such primary screening processes because (i) naïve CD4T cells are the major population that reacts against allogeneic antigen presenting cells, indicating that the cytokines detected in the cell-culture supernatant fluids of MLR are of newly differentiated CD4T cells; (ii) it is mediated by the physiological MHC-peptide-TCR interactions; (iii) the clonal frequency of alloreactive naïve CD4T cells is markedly higher than that of foreign antigen-specific naïve CD4T cells; and (iv) alloreactive Th1, Th2, and Th17 cells are induced without the use of externally added cytokines, allowing us to target all the Th subsets in a single cell-culture well. Using these systems, we revealed that the administration of a D1-like-R antagonist inhibits Th17 differentiation, thereby improving clinical phenotypes, including experimental autoimmune encephalomyelitis (EAE), type 1 diabetes, glomerulonephritis, rheumatoid arthritis [8], and neutrophilic airway inflammation [9], in mouse models.

In order to identify novel compounds that have inhibitory effects on Th17 generation, we focused on a traditional Japanese herbal medicine, Kampo, which is well known to exert various immunomodulatory effects [10–12]. In this report, we screened several Kampo extracts known to possess anti-inflammatory activities and identified two Kampo extracts with Th17-inhibitory activity. One is oren-gedoku-to (OGT), which is ethically prescribed to treat inflammatory diseases, including dermatitis, gastric ulcers, gastritis, and related diseases. The other is hange-shashin-to (HST), which is effective in treating disorders of the digestive mucosa, such as gastritis, diarrhea, and stomatitis. OGT and HST have been studied in several experimental models of gastritis and colitis [13–16]; however, their mechanisms have yet to be determined. Both OGT and HST contain common components originating from two kinds of medicinal herbs, Scutellaria root and Coptis rhizome. A representative flavonoid, wogonin, is one of the primary components of Scutellaria root, and berberine is an isoquinoline alkaloid that is rich in Coptis rhizome. The aim of this study is to identify the active components contained in OGT and HST and to evaluate their immunomodulatory features, particularly with respect to T-cell lineage differentiation.

## 2. Materials and Methods

### 2.1. Test Samples

Kampo medicines are aqueous extracts of a mixture of natural crude drugs. Eight kinds of Kampo extracts, HST, OGT, HJG (hachimi-jio-gan), SST (sho-saiko-to), HET (hochu-ekki-to), JTT (juzen-taiho-to), DKT (dai-kenchu-to), and SRT (sai-rei-to) were obtained from Tsumura and Co. (Tokyo, Japan) in the form of dried powdered extract. Berberine and wogonin were purchased from Wako Pure Chemical Industries, Ltd. (Osaka, Japan). Alternatively, wogonin was extracted from Scutellaria root at Tsumura and Co. at a purity of more than 99.5% for the* in vivo* studies.

The Kampo extracts were suspended in dimethyl sulfoxide (DMSO) at 10 mg/mL and passed through a 0.22 *μ*m membrane following gentle centrifugation. The supernatant fluids were added to the cultures at a final concentration of 10 *μ*g/mL. The wogonin and berberine were dissolved in DMSO and added to the cultures at the described concentrations.

### 2.2. Mice

SJL and BALB/c mice were obtained from Charles River Japan, Inc. (Kanagawa, Japan). OVA TCR-transgenic DO11.10 mice were obtained from the Jackson Laboratory (Bar Harbor, ME). All animal experiments were approved by and performed in compliance with the Institutional Animal Care and Use Committee Guidelines.

### 2.3. Preparation of Human Mo-DCs and T Lymphocytes

Human Mo-DCs and CD45RA^+^ naïve CD4^+^ T cells (>99% purity) were prepared as previously described [9]. Briefly, human Mo-DCs were prepared from human peripheral blood specimens (PBMCs) using positive selection with CD14 MicroBeads (Miltenyi Biotec). The Mo-DCs were then cultured in the presence of GM-CSF (50 ng/mL) and IL-4 (50 ng/mL) for five days. Human CD45RA^+^ naïve CD4^+^ T cells were prepared via negative selection using a naïve CD4^+^T-cell isolation kit II (Miltenyi Biotec, Auburn, U.S.A.). The study using PBMCs obtained from healthy volunteers was approved by the Saitama Medical University Ethics Committee.

### 2.4. Two-Way MLR Assay

For the mouse two-way MLR, 3 × 10^6^ spleen cells obtained from SJL/J mice (H-2^s^) and 3 × 10^6^ spleen cells obtained from BALB/c mice (H-2^d^) were mixed and then incubated with Kampo extracts (10 *μ*g/mL), wogoinin (0.1, 0.3, and 1 *μ*M), or berberine (0.1, 0.3, and 1 *μ*M) in 2 mL of DMEM medium containing 10% FCS in a flat-bottomed 12-well plate for seven days. The culture supernatants were assayed for IFN-*γ*, IL-4, IL-5, IL-6, and IL-17 using ELISA kits (R&D systems, U.S.A.). For the human two-way MLR, 3 × 10^6^ human PBMCs were incubated with 3 × 10^6^ HLA-DR nonshared allogeneic PBMCs in the presence or absence of wogonin (1 *μ*M) in RPMI 1640 medium supplemented with 10% human serum in a flat-bottomed 12-well plate for seven days. The culture supernatants were assayed for IFN-*γ*, IL-4, IL-5, and IL-17 using ELISA kits (R&D systems, U.S.A.).

### 2.5. DC-Mediated T-Cell Differentiation Assay (One-Way MLR)

For the human one-way MLR, 1 × 10^4^ human immature Mo-DCs were seeded in a round-bottomed 96-well plate and then incubated in the presence or absence of wogonin (1 *μ*M) in 100 *μ*L of RPMI 1640 medium containing 10% human serum for two days. Following incubation, the Mo-DCs were washed once and then cocultured with 1 × 10^5^ HLA-DR nonshared allogeneic CD4^+^ naive T cells in RPMI 1640 medium supplemented with 10% human serum in a round-bottomed 96-well plate for seven days. At seven days after incubation, the cells were washed twice and stimulated with anti-human CD3 and anti-human CD28 antibodies (BD Pharmingen, San Diego, U.S.A.) for 24 h. The supernatant was then collected for the IFN-*γ*, IL-4, IL-5, and IL-17 ELISA.

### 2.6. Induction of Airway Inflammation and Administration of Wogonin

Six- to ten-week-old female DO11.10 mice were challenged with an aerosolized solution of 3% OVA (Sigma-Aldrich, St. Louis, U.S.A.) or PBS for 10 min from days −2 to 0. From days −7 to 0, some mice received wogonin (2 mg/kg/day; Tsumura) in distilled water (100 *μ*L) or the vehicle orally every day. The mice were sacrificed, and the degree of neutrophilic airway inflammation was evaluated on day 1.

### 2.7. Bronchoalveolar Lavage Fluid (BALF) Analyses

The BALF analyses were performed as previously reported [12]. The mice were anesthetized via intraperitoneal injection of sodium pentobarbital (50 mg/kg). Then, the lungs were lavaged four times with PBS (0.5 mL each). Approximately 1.6 mL of the instilled PBS was consistently recovered with gentle handling. The cell suspension was centrifuged at 150 ×g at 4°C for 10 min. The cells were resuspended in 1 mL of PBS, and the total number of cells was counted with a hemocytometer. Cytospin samples were prepared by centrifuging the suspensions at 300 rpmfor 10 min. Based on the findings of May-Grünwald-Giemsa staining, the cell differentials were counted with at least 300 leukocytes in each sample. The cell types were judged according to standard hemocytologic procedures to be either neutrophils, eosinophils, lymphocytes, or macrophages.

### 2.8. Histological Examination

The histological examination was performed as previously reported [9]. Following perfusion with PBS, the right lungs were resected, fixed with 10% neutralized buffered formalin (Wako, Osaka, Japan), and embedded in paraffin. Three-micrometer-thick sections were stained with hematoxylin and eosin.

### 2.9. Cytokine Production and Proliferation Assay of Human Th17 Clones

Human alloreactive Th17 cell clones established in our previous study were used [17]. To analyze the cytokine production, Th17 cells were cocultured with irradiated allogeneic PBMCs in medium containing RPMI 1640 and 10% human serum and then were incubated for 24 h in the presence or absence of 1 *μ*M of wogonin. Following incubation, the supernatants were analyzed using an IL-17 ELISA. To assess the proliferation of the human Th17 clones, the cells were incubated with irradiated allogeneic PBMCs in the presence or absence of 1 *μ*M of wogonin for 72 h in the presence of 1 *μ*Ci/well of [^3^H] TdR during the final 16 h period, and the incorporated radioactivity was measured using liquid scintillation counting after harvesting.

### 2.10. Statistics

The values are expressed as the mean ± SEM. The statistical analyses were performed using a one-way ANOVA followed, when the differences were significant, by appropriate post hoc tests with the Tukey-Kramer test. To analyze differences between the two groups, we used Student's *t*-test. Values of *P* < 0.05 were considered to be statistically significant.

## 3. Results

### 3.1. Screening of Kampo Extracts to Identify the Inhibitory Effects on Th17

**Figure 1:**
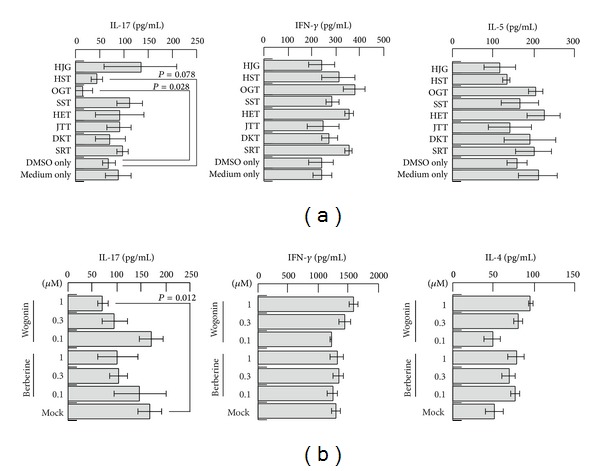
Screening of Kampo extracts and identification of major components that exhibit the inhibitory effects of IL-17 secretion. HJG, HST, OGT, SST, HET, JTT, DKT, or SRT were added at a concentration of 10 *μ*g/mL to the two-way MLR of splenic lymphocytes obtained from BALB/c and SJL mice and then were incubated for seven days (a). The supernatants were analyzed using ELISA to detect the typical cytokine secretions of IFN-*γ* (*center*), IL-5 (*right*), and IL-17 (*left*) for Th1, Th2, and Th17, respectively. Increasing concentrations of wogonin or berberine (0.1, 0.3, and 1 *μ*M) were mixed with a culture of lymphocytes obtained from BALB/c and SJL mice and then incubated for seven days (b). The supernatants were analyzed using ELISA to detect the typical cytokines of IFN-*γ* (center), IL-4 (right), and IL-17 (left) for Th1, Th2, and Th17, respectively.

In order to identify the compounds in the Kampo extracts that attenuate IL-17 production during the immune response, eight Kampo extracts were subjected to mouse two-way MLR, and the concentrations of the cytokines secreted during the immune reactions were evaluated. For this purpose, spleen cells obtained from BALB/c and SJL mice were mixed in the presence or absence of the Kampo extracts, and seven days after the initiation of the culture, the supernatants were collected and subjected to ELISA for cytokines. As shown in Figure 1(a), OGT significantly suppressed the IL-17 production. Although the *P* value was more than 0.05, HST also reproducibly suppressed the IL-17 production. The experiments were repeated three times, and the same results were obtained in each experiment. Wogonin and berberine are the major components common to the two Kampo extracts and were therefore evaluated in the further assays.

### 3.2. Activity of Wogonin in the Mouse Two-Way MLR

We therefore evaluated the activity of wogonin and berberine during the immune reactions in the mouse two-way MLR (Figure 1(b)). As expected, wogonin significantly suppressed the IL-17 production at a concentration of 1 µM. Although the effects were lower in magnitude, berberine also suppressed the IL-17 production at a concentration of 1 µM. The experiments were repeated twice, and the same results were obtained each time.

### 3.3. Activity of Wogonin in the Neutrophilic Airway Inflammation Model

**Figure 2:**
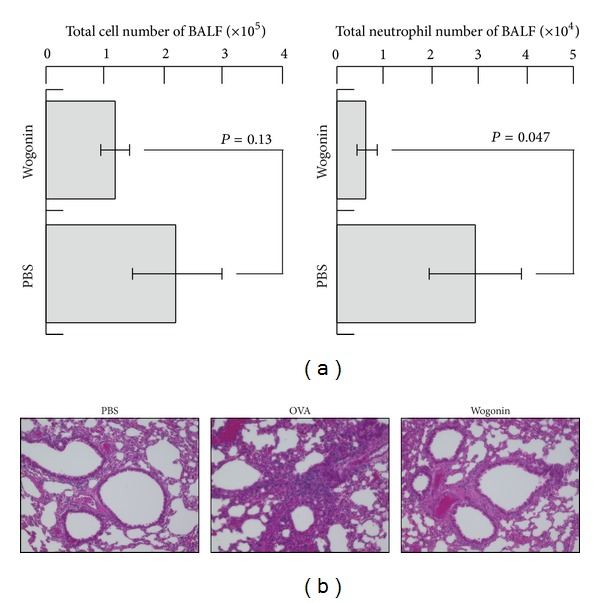
Decline in the number of neutrophils infiltrating the bronchi and lungs induced by wogonin treatment. DO11.10 mice were nebulized with either OVA or PBS from days −2 to 0. Some mice received wogonin (2 mg/kg) or distilled water orally five times a week from days −7 to 0. On day 1, the mouse BALF was collected in order to count the number of total cells ((a),* left*) and neutrophils ((a),* right*). The mouse lungs were excised and subjected to hematoxylin and eosin staining (b). Histological staining was performed on the lungs removed from the mice that received water and were nebulized with PBS (left panel), the mice that received water and were nebulized with OVA (center panel), and the mice that received wogonin and were nebulized with OVA (right panel). Original magnification: ×200.

To elucidate the effects of wogonin* in vivo*, an OVA-induced neutrophilic airway inflammation model using DO11.10 mice was employed [9]. The administration of wogonin was performed starting six days before OVA nebulization. OVA nebulization markedly increased the number of neutrophils in the BALF of the DO11.10 mice, while the increase in the level of neutrophils was significantly suppressed by wogonin treatment (Figure 2(a)). The histology of the OVA-challenged DO11.10 mice demonstrated marked congestion and prominent neutrophil infiltration in the peribronchial areas (Figure 2(b)). In contrast, the degree of infiltration was markedly lower in the mice that received wogonin. The experiments were repeated twice, and the same results were obtained each time. These results suggest that wogonin has an effect on neutrophil-mediated airway inflammation via Th17 suppression.

### 3.4. Effects of Wogonin in the Human MLR

**Figure 3:**
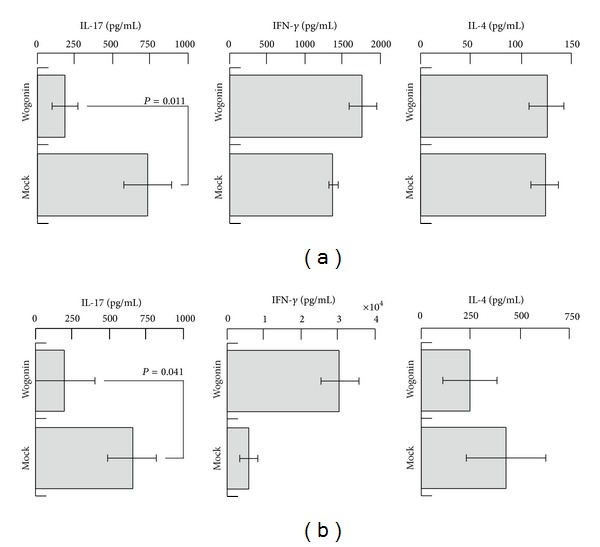
Suppression of IL-17 by wogonin treatment in the human MLR. For the human two-way MLR (a), wogonin (1 *μ*M) was exposed to a mixture of human allogeneic lymphocytes and then incubated for seven days. Seven days after incubation, the supernatants were analyzed using ELISA to detect the typical cytokines of IFN-*γ* (center), IL-4 (right), and IL-17 (left) for Th1, Th2, and Th17, respectively. For the human one-way MLR (b), wogonin (1 *μ*M) was incubated with human Mo-DCs for two days. Then, the wogonin-treated human Mo-DCs were incubated with human allogeneic naïve CD4^+^T cells in the absence of wogonin for seven days. The cells were restimulated with anti-human CD3 and CD28 antibodies for 24 h, and the supernatants were collected for ELISA to detect the typical cytokines of IFN-*γ* (center), IL-4 (right), and IL-17 (left) for Th1, Th2, and Th17, respectively.

To evaluate the effects of wogonin on the human immune system, a human two-way MLR was performed (Figure 3(a)). Again, wogonin exhibited suppressive activity against IL-17 production. The production of IL-4 was not significantly affected. These observations collectively indicate that wogonin suppresses the IL-17 production during immune responses in both mouse and human systems, raising the possibility that wogonin inhibits DC-mediated Th17 differentiation. To address this issue, wogonin was subjected to a human one-way MLR (Figure 3(b)). Human monocyte-derived immature DCs were incubated with wogonin for two days and then were incubated with human allogeneic naïve CD4^+^T cells in the absence of wogonin. Seven days after incubation, the cells were stimulated with anti-human CD3 and anti-human CD28 antibodies for 24 h. The supernatant was then collected and analyzed using ELISA to assess the production of IL-17, IFN-*γ*, and IL-4. As expected, the production of IL-17 was suppressed by the treatment of immature DCs with wogonin, suggesting that wogonin inhibits DC-mediated Th17 differentiation.

### 3.5. Wogonin Does Not Inhibit the Secretion of IL-17 or the Proliferation of Th17 Cells

**Figure 4:**
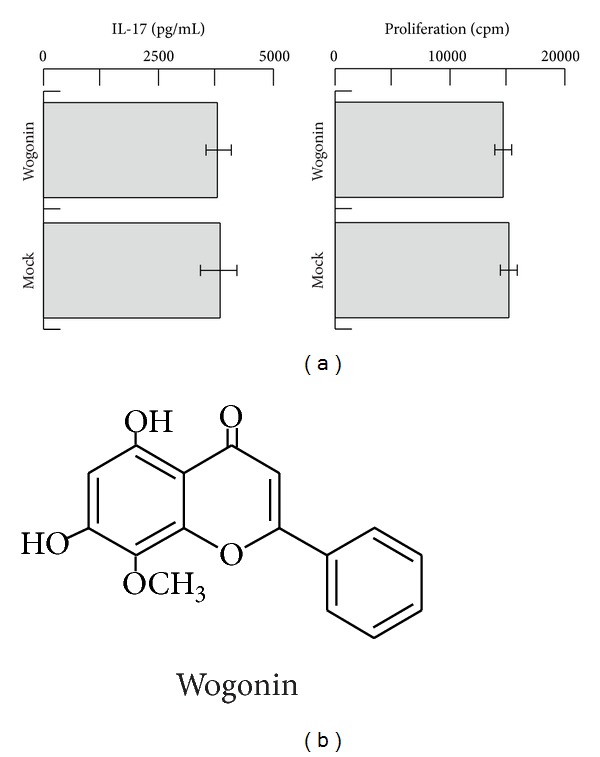
Effects of wogonin on a human Th17 clone. The human alloreactive Th17 clone was established earlier [17]. Following incubation of Th17 with irradiated allogeneic PBMCs in the presence or absence of wogonin (1 *μ*M) for 24 h, the supernatants were analyzed using ELISA to detect the secretion of IL-17 ((a), left). To assess the proliferation of Th17, the cells were incubated with irradiated allogeneic PBMCs in the presence or absence of wogonin (1 *μ*M) for 72 h and analyzed using [^3^H] TdR incorporation during the final 16 h period. The incorporated radioactivity was analyzed using liquid scintillation counting after harvesting ((a), right). Wogonin belongs to flavonoid species (b).

In order to exclude the possibility that wogonin directly inhibits the production of IL-17 and proliferation of Th17 cells, cloned human Th17 cells established in our previous study [17] were cocultured with wogonin (Figure 4(a)). Consequently, neither IL-17 production nor proliferative responses were affected. The experiment was repeated once, and the same results were obtained. All of these results collectively indicate that wogonin does not affect activated Th17 cells, but rather abrogates the DC-mediated differentiation of naïve T cells to Th17 cells.

## 4. Discussion

Recently, accumulated data on immunological disorders have indicated that the generation of Th17 is closely associated with the development of many autoimmune conditions [18]. Since IL-17 induces chemoattraction of neutrophils, the activation of Th17 for IL-17 secretion is speculated to exacerbate the neutrophilic inflammation observed in many autoimmune diseases. It is well known that several Kampo medicines exhibit efficacy in suppressing inflammation, including that observed in gastritis, colitis, arthritis, hepatitis, pneumonia, and dermatitis. However, the detailed mechanisms underlying the anti-inflammatory effects of these Kampo medicines remain largely unknown. We presumed that the compounds contained in Kampo extracts would inhibit Th17 generation. In order to identify such molecules, we decided to use our established systems to analyze the Th1-Th2-Th17 balance and adjuvant activities.

Eight kinds of Kampo extracts were initially examined in our screening assay to identify which Kampo extracts are capable of inhibiting the IL-17 production. OGT significantly reduced the allogeneic antigen-induced production of IL-17, but not that of IL-4 or IFN-*γ*. Moreover, HST also showed a tendency to reduce IL-17, although the inhibitory effect of HST was less than that of OGT. OGT is an extract of a mixture of the following four medicinal herbs in the ratios provided in the parentheses: Scutellaria root (3.0), Coptis rhizome (2.0), Gardenia fruit (2.0), and Phellodendron bark (1.5). HST is an extract of a mixture of the following seven medicinal herbs in the ratios provided in the parentheses: Scutellaria root (2.5), Coptis rhizome (1.0), Pinellia tuber (5.0), Processed ginger (2.5), Glycyrrhiza (2.5), Jujube (2.5), and Ginseng (2.5). Therefore, both OGT and HST have common components originating from Scutellaria root and Coptis rhizome. In addition, wogonin and berberine are more abundant in OGT than in HST because the constitutive ratios of the two common medicinal herbs in OGT are higher than those observed in HST. The other six kinds of Kampo extracts that demonstrated no effects on the IL-17 production do not contain Coptis rhizome. The SST and SRT extracts contain Scutellaria root, although at an extremely lower amount than OGT or HST, considering the constitutive ratios of the two medicinal herbs. The present study indicates that wogonin and berberine exhibit strong and selective activities to inhibit the allogeneic antigen-induced IL-17 production. Furthermore, we revealed that wogonin suppressed DC-mediated Th17 differentiation in a human one-way MLR. On the other hand, in the human Th17 clone, wogonin had no effect on the secretion of IL-17 or proliferation. Overall, the results suggest that wogonin affects DCs rather than already-differentiated Th17 cells in the suppression of IL-17 secretion.

Previously, a dopamine D1-like-R antagonist, SCH23390, was found to significantly improve neutrophilic airway inflammation induced by OVA administration [9], suggesting that the inhibitory activity of wogonin against D1-R-mediated signaling may also improve neutrophilic airway inflammation. We therefore attempted to show the activity of wogonin by using D1R antagonism assay, D1R binding assay, and tyrosine hydroxylase inhibition assay. However, all these experiments gave negative results (not shown).

Wogoin belongs to flavonoids (Figure 4(b)). In this relation, Morita, et al. demonstrated that other flavonoids inhibited the release of catecholamines [19], which should also be tested with wogonin. It is also possible to speculate that wogonin may interact directly with naive T cells, because both DCs and T cells are exposed to wogonin, in two-way MLR assay shown in our current study. In this case, molecules such as Akt, mTOR, HIF-1*α*, and STAT3 expressed in naive T cells need to be considered as targets for wogonin [20]. The study is currently underway.

HST and OGT have classically been shown to harbor anxiolytic effects, including neurosis and insomnia. From this point of view, wogonin also has been shown to exert anxiolytic effects in the screening of components of HST and OGT [21]. In parallel, Kampo extracts have an effect on cancer prevention [22, 23], and wogonin has been clarified to have an antitumor effect in bladder cancer cell lines [24]. Additionally, wogonin inhibits the glioma-mediated induction of the Treg phenotype via inhibition of the TGF-*β*1 activity [25]. Immunomodulatory effect of wogonin on virus infection [26] and LPS stimulation [27] has been observed; however, the effects of wogonin on Th1-Th2-Th17 balance have not been considered to date. This is the first report, to our knowledge, to show that wogonin has the potency to reduce the Th17 population, which should greatly contribute to the effective treatment of autoimmune disorders. Although increasing evidence has shown the cytotoxicity of wogonin in multiple cancer cell lines [28, 29], cell viability was not affected by coculture with wogonin in MLR experiments (data not shown).

## 5. Conclusions

Taken together, the findings of the current study demonstrated that wogonin has the potency to selectively suppress DC-mediated Th17 differentiation. Our study suggests that wogonin may, therefore, be an effective therapeutic agent for Th17-driven diseases and its use may thus be useful as a new strategy to treat neutrophilic inflammatory disorders.
